# Breastfeeding knowledge of mothers in protracted crises: the Gaza Strip example

**DOI:** 10.1186/s12889-021-10748-2

**Published:** 2021-04-17

**Authors:** Iellamo Alessandro, Emily Monaghan, Samar A. L. Moghany, Jonathan Latham, Nihal Nassereddin

**Affiliations:** 1 Save the Children, London, UK; 2 Save the Children International, Occupied Palestine Territory, Gaza, Palestine; 3 World Food Programme Palestine, Jesuralem, Israel

**Keywords:** Breastfeeding, Breastfeeding in emergencies, IYCF, IYCF-E, New-born care, Early initiation of breastfeeding, Infant feeding in emergencies, Midwives, Counselling, BFHI

## Abstract

The protection and support of breastfeeding is the most effective intervention to prevent child morbidity and mortality especially in humanitarian crisis.

During the Palestine-Israel conflict healthcare services are understaffed and lack basic resources, with frequent power cuts and stock-outs of essential drugs and equipment. This case study seeks to answer the questions: (1) How does the protracted crisis in Gaza affect the breastfeeding practices of the most vulnerable population; and (2) What is the role that midwives can play in improving breastfeeding practices?

The study was conducted using a mixed method approach with quantitative and qualitative methods. Purposeful selection of women and children was conducted utilising eligibility criteria, women with children less than 2 years of age were included. All the respondents were asked if they agreed to participate in the survey.

A total of 63% practice early initiation of breastfeeding and 42% confirmed that their new-borns were given liquids other than breast milk during the first 3 days of life. Fifty percent of mothers addressed breast milk insufficiency by drinking additional fluids and 40% by using infant formula. Only 18% of women said that they received breastfeeding information during contact with health professionals throughout labour, delivery, and subsequent post-natal care visits. Many mothers during the focus group discussions (FGDs) confirm using milk to top up or replace breast milk.

Myths and misconceptions around breastfeeding remain, while women do access antenatal care services and deliver in the health facilities. There is a need to a) adapt the recommendations of the operational guidance for infant and young child feeding in emergencies (IYCF-E) in the Gaza strip, to protect, promote and support breastfeeding and b) include skilled breastfeeding counselling in the pre-service and in-service training for midwives.

Lessons learned included the importance of a) allocating additional research time, to account for interruption b) daily coordination with security officers to ensure safe access to localities c) identification of extra sites, in case of conflict escalation d) training of additional enumerators in case conflict escalation e) negotiation with authorities to ensure compliance with requirements.

## Background

### Humanitarian context

Sub-optimal breastfeeding is associated with more than 800,000 under 5 years old deaths annually worldwide [1]. Exclusive breastfeeding for the first 6 months and continual breastfeeding may prevent 13% of under-five deaths, primarily from infections resulting in diarrhea, pneumonia, and neonatal sepsis [2]. Beyond the first years of life, breastfeeding has been found to improve children’s quality of life by preventing various diseases such as leukemia, asthma, ear infections, allergies, and diabetes [3, 4]. Recent systematic reviews have highlighted the role that midwives have in the protection, promotion, and support of breastfeeding, [5]. The findings show that midwives value breast feeding education and breastfeeding support but the way they provide it varies across contexts [5]. The same review suggests that the hospital-based midwives face more challenges like time constraints [5], A Cochrane review, confirms that providing women breastfeeding support, helps ensure longer breastfeeding [6]. The same review suggest that the breastfeeding support is more effective when the support is predictable, scheduled, and includes regular visits to health professionals like midwives [6].

In the Gaza Strip, the health sector has been heavily disrupted by years of conflict, sanctions, and socio-economic decline [7]. Healthcare services are understaffed and lack basic resources, with frequent power cuts and stock-outs of essential drugs and equipment [8–10]. Over 90% of the water is unsuitable for human consumption [8–10]. Psychological trauma and poverty have severely affected the population’s mental health, with many people, including children and pregnant and lactating women, suffering from anxiety and depression [9, 10]. In 2014 the prevalence of early initiation of breastfeeding dropped drastically from 66 to 41% [11]; MICS 2014, revealed very low level of exclusive breastfeeding (36.5%) (64th of 133 countries), and that only less than 60% of children are breastfed up to 1 year and the same drops to a 10% of children are breastfed up to 2 years [10]. During crisis and emergencies like the Gaza strip, the protection, promotion, and support of breastfeeding is a lifesaving and food security intervention that needs to be supported [3, 4].

As a part of a larger multisectoral assessment, this paper considers how the protracted crisis in Gaza has affected breastfeeding practices of the most disadvantaged women. It investigates (1) How does the protracted crisis in Gaza affect the breastfeeding practices of the most vulnerable population; and (2) What is the role that midwives can play in improving breastfeeding practices?

## Main text

### Research study

#### Methods

This case analysis highlights a component of a larger nutrition multisectoral assessment conducted in the Gaza strip and focuses on the following questions:

1. How does the protracted crisis in Gaza affect the breastfeeding practices of the most vulnerable population;
2. What is the potential role that midwives can play in improving breastfeeding practices?

##### Design

A mix method was used to conduct the assessment in nine of the most vulnerable localities across the five governorates of the Gaza strip. Most vulnerable populations are defined as being the poorest and with the lowest educational attainment [12]. The 2018 data provided poverty information at the governorate level and not at the locality level, so several proxy indicators were used to determine the poverty levels, and this included the household size, highest educational attainment, and female literacy levels [12].

The household survey included the following components: a) anthropometric measurements for children 6–59 months, b) MUAC measurement for pregnant women and mothers of children less than 6 months c) an IYCF survey d) focus group discussions with primary caregivers and e) key informant interviews with stakeholders.

##### Sample

The sample size was estimated considering statistical confidence for each of the breastfeeding indicators.

##### Data collection

The household questionnaire was developed using Excel, the same was then uploaded into an online tool (Kobo). Once the question was uploaded in Kobo, it was shared with the assessment team for review, testing and finalization.

The questions about breastfeeding were asked only to the primary caregivers with children less than 2 years [13]. The questions related to breastfeeding practices included the time of initiation of breastfeeding, current breastfeeding practices, access to breastfeeding information, including the source of support when experiencing breastfeeding problems [13].

A total of 22 female enumerators living in the Gaza strip and with previous nutrition experience, were recruited. The enumerators were divided in 11 teams of 2 members each and the lead agencies provided them with a 3-day training [13].

The conduct of the assessment was approved by the Ministry of Interior in the Gaza strip. The Ministry of Interior reviewed and provided comments to the questionnaire and the methodology, including the text used to seek the consent of the respondents [13].

During the data collection the enumerators used systematic random sampling to select the households to be included in the survey. At the household level women were selected purposefully, using the pre agreed selection criteria. A household was eligible for the interview if there was a child < 2 years of age and the primary caregiver was present [13]. Verbal consent to participate was asked to all the target respondents and was recorded in the Kobo tool. A total of 1476 respondents (primary caregivers) of children (0–59 months) were covered by the assessment but only 1044 had a child less than 24 months (70.7%) while the remaining had children 2 to less than 5 years of age [13].

During the data collection phase, two (2) initially selected areas could not be accessed due to rapid deterioration in the security situation and associated risks of conflict escalation with aerial attacks from both parties involved. Data collection was therefore interrupted for 2 days due to the imminent risk of escalation between the parties. Clearance was provided by the security officer prior to the re-start of data collection [13].

A total of four focus groups discussions (FGDS) were conducted to better understand the local beliefs and other factors affecting infant feeding practices [13]. The FGDs targeted only women and were conducted in two clinics in the city of Gaza and Khan Yunis within Gaza strip, with a total of 5 women from each of the localities covered by the household survey [13]. The participants to the FGD were selected from the respondents of the household survey based on their responses to the breastfeeding questions. For each of the FGDs half of the participants had good breastfeeding practices while the other half had poor breastfeeding practices. The selection of the two type of participants helped barriers and facilitators to breastfeeding [13].

##### Data analysis

The completed questionnaires were downloaded from the Kobo cloud onto Excel, this was done to clean and translate all the data entries in English [13]. The anthropometric data were exported into the ENA Smart Software, for the calculation of the different anthropometric indicators and to conduct the regular plausibility check [13]. The food security information was processed and analysed with the support of WFP technical team of the Jerusalem office. All the remaining data were process and analysed using SPSS [13].

## Results

A total of 1476 respondents (mothers) of children (0–59 months) were covered by the assessment, and all (100%) delivered in a health facility. 1172 (80.7%) delivered vaginally, while 284 (19.2%) delivered via a Caesarean-section (CS). A total of 1044 had a child less than 24 months (70.7%) while the remaining had children 2 to less than 5 years of age.

The study revealed that 6.3% of mothers with children less than 24 months stated that they never breastfed their children. Early initiation of breastfeeding (breastfeeding within the 1st hour of birth) was practiced by 62.75% of mothers of children aged 0–23 months; 42% of the respondents confirmed that their new-borns were given liquids other than breast milk during the first 3 days of life.

Findings show that in the context of the Gaza strip, 88% of women know that breastfeeding should be initiated within the first hour of birth (Table 1).

**Table 1:** Knowledge on when to start breastfeeding

When should you start breastfeeding?	No	(%)
Immediately/within 1 hour	919	87.77
Within 1 day	87	8.31
Within 2 days	23	2.20
When the mother is ready	12	1.15
When the baby wants	3	0.29
Don’t know	2	0.19
After 3 days	1	0.10

More than 50% of women said that they received most breastfeeding information during antenatal care visits. Only 18% of women said that they received breastfeeding information during contact with health professionals throughout labour, delivery, and subsequent post-natal care visits.

When asked about the reasons for never breastfeeding their children, the top five (5) reasons given were: 1) maternal illness (40.3%); 2) new-born illness (26.9%); 3) baby’s refusal to breastfeed (16%); 4) perceived no/insufficient breast milk (9%); and 5) preterm baby (4.5%) or CS delivery (4.5%).

Mothers were asked about the way that they addressed breastfeeding complications, such as perceived breast milk insufficiency. More than 50% answered that they rely on drinking additional fluids, 40% started to use breast milk substitutes such as infant formula, 21% of women increased the frequency of breastfeeding and 3% sought counselling support (Table 2).

**Table 2:** Respondents’ solutions to perceived breast milk insufficiency

What should you do if you have insufficient breast milk?)	No.	(%)
Drink more fluids	533	51%
Top up each breastfeed with a bottle of formula	431	41%
Increase frequency of breastfeeding	220	21%
Unsure / Don’t know	141	13.5%
Seek advice/assistance with positioning and attachment	37	3.5%

Focus group discussions conducted with women affected by the conflict confirm quantitative findings. Qualitative findings show a very high awareness of recommended breastfeeding practices, but the concerns and worries about the current economic situation, coupled with misinformation currently affect the breastfeeding practices in these communities. Some of the mothers of infants less than 6 months attending the FGDs said that they are exclusive breastfeeding.

*“It is better to commit to exclusive breastfeeding rather than supplementary food we cannot afford”*, one woman said.

Other women said that “*they do not have enough breastmilk, so they have to resort to infant formula, yogurt or cerelac to help the child feeding beside the not enough breastfeeding”*. One woman said that she gives child formula because she has twins and another one because her health is not good to give enough breastfeeding for child.

## Discussion

### Scientific importance of this research

The findings illustrate the level of breastfeeding knowledge and practices of mothers living in poor communities, suggesting an opportunity to increase the ability of the midwives in the Gaza strip to promote, protect, and support breastfeeding during ante-natal, intra-partum and the post-natal period. This assessment provided new, updated information on the breastfeeding practices of the most vulnerable communities in the Gaza strip. Prior to this study the most recent data was the Multiple Indicator Cluster Survey of 2014 [11].

Breastfeeding saves lives in all contexts - even more so in humanitarian and fragile contexts. The protection, promotion and support of breastfeeding are among the most effective interventions to prevent child morbidity and mortality in humanitarian contexts [2]. Exclusive breastfeeding for the first 6 months and continued breastfeeding, thereafter, may prevent 13% of under-five deaths, primarily from infections resulting in diarrhoea, pneumonia, and neonatal sepsis [2]. Non breastfed infants have a 14 times higher risk of dying from any cause compared to exclusively breastfed infants, a 15 times higher risk pneumonia and a 10 times higher risk of diarrhoea [14, 15]. Furthermore, the global impact of breastfeeding on maternal health is significant, with a reduction in the risk of breast cancer and potential to prevent 20,000 deaths [1]. In environments where access to safe water is limited and food insecurity is a significant issue for families, exclusive breastfeeding for new-borns and infants is a lifesaving public health intervention [14–21]. It is important to ensure that additional studies are conducted in similar contexts, to ensure that findings are reflected in practical measures to protect the lives of the youngest and most vulnerable in humanitarian settings, whilst providing the needed support for pregnant women and mothers.

This study’s findings highlight the potential role that midwives in humanitarian contexts, who have an integral role in the initiation, protection promotion and support of breastfeeding practices. The findings were disseminated and validated by the members of the Nutrition Working Group and were presented to national and international organizations working in the Gaza strip during a workshop in May 2019.

The same were also presented to the different sector’s coordination mechanism including Health, WASH, Food Security, Livelihoods and Protection from June to August 2019.

The findings illustrate the immediate need to focus on practical, focussed interventions aiming at the most vulnerable were shared and presented with the Humanitarian Country Team (lead by UN OCHA), UN agencies and the organizations and donors contributing and supporting the humanitarian response. This guided strategic discussion and initial direction with UN OCHA and relevant UN partners. The study suggests that the protection, promotion, and support of breastfeeding focusing on the most vulnerable populations in the Gaza strip, will receive more attention, including financial resources. The findings of the study were used to inform the revision and update of the Humanitarian Needs Overview for 2020 and the Humanitarian Response Plan 2019–2020 providing additional windows of opportunity for resources for an integrated solution to the issues presented.

Evidence shows that when breastfeeding support is offered to women, the duration of exclusive breastfeeding is increased [6]. Studies have confirmed the benefits that individual breastfeeding support provided by midwives have on women and their practices [16, 22, 23]. This is particularly relevant in conflict settings, where the need for psychosocial and mental support for pregnant and postnatal women with babies is significant, given the continuous challenge of giving birth and raising children in such a difficult and unsafe environment.

The findings confirm the need to: a) ensure a concerted effort to fully adapt and implement the recommendations of the IYCF-E operational guidance [24]; b) review and possibly improve the pre-service training for midwives and other health professionals who provide antenatal, intrapartum and post-natal care in relation to the protection, promotion and support of breastfeeding using recommended WHO modules [25]; and scale up and exert efforts to ensure hospital and health facilities providing maternity services fully comply with the WHO/UNICEF Baby Friendly Hospital Initiative [26].

For the vulnerable population of the Gaza strip, it is imperative to support the midwives to provide skilled support at birth (both vaginal and caesarean) thus ensuring breastfeeding initiation. If breastfeeding is practiced in the Gaza strip as per recommendations, there will be increased protection for common childhood diseases such as diarrhoea and ARI, whilst ensuring that the available resources are used by the household to increase access to a diverse and appropriate diet for the mother and the other members of the family. Having skilled midwives providing lactation support and counselling is certainly recommended in all contexts, particularly humanitarian settings, and fragile environments like the Gaza strip.

### Challenges to research

#### Security

The security situation in the Gaza strip is unpredictable and escalation of conflicts is always a possibility. This was a major consideration during the design and planning phase of the study, and plans were made to ensure successful completion, even in the event of potential security situations. Contingency plans included the identification of back up areas to be covered and/or the reduction of the sample size requirements were also included.

#### Political situation

The current political situation in the Gaza strip, exemplified by a strict set of requirements limiting study coverage and information to be collected by the Ministry of Interior in Gaza, was a critical challenge faced during the study. The Ministry of Interior reviewed the proposed questionnaire, as per local policy and requested that questions on socio-economic status and mental health be removed. This did not allow for an analysis of the results linking infant feeding practices and psychological condition of the caregiver, which, in a context like the Gaza strip, could be remarkable.

#### Capacity

Due to the conflict and travel restrictions within the Gaza strip, many professionals and health workers (including midwives) are not able to access regular trainings and capacity building opportunities. This is clearly identified as a major gap and highlighted the need to strengthen local capacity in relation to nutrition assessments and implementation using local capacities and resources. This is relevant to this study’s findings, with associated challenges when finalising study design and methodology, data collection and the quality of some aspects of the study.

#### Research strategies

The following strategies and actions were deployed to address most of the challenges encountered:

- *Integration and linkage with security officers* of leading organizations was essential, during the design, planning and execution of all the activities. During the data collection phase, it was continuous and provided valuable information that led to the temporary interruption of the data collection due to a rapid escalation of the conflict affecting the selected areas. In a volatile and unpredictable context like the Gaza strip, daily movements of teams and large groups must be cleared by relevant officers, mostly considering the locations of destination.
- *Coordination with authorities and communities*; a requirement for the issuance of the needed formal endorsements and clearances by the governing authorities as well ensuring full engagement and support from the participating communities. The Ministry of Interior in Gaza (under the Hamas leadership) required the review and clearance of the design and questionnaires. Several questions were removed as per their recommendations, that called for a revision of the questionnaire and of the objectives of the study.
- *Supportive supervision* was provided during the whole data collection with daily debriefing with teams, with the support of the leading organizations and to the extent possible international consultant. However, considering the escalation of the hostility during this period, this was reduced drastically and limited to Phone SMS based follow ups and updates. Teams were asked to communicate locations, challenges, and progress, regularly during the day, to ensure their safe and smooth progress in the data collection.
- *Allocation of buffer days and geographical areas in lieu of days lost*. Having a contingency plan for similar exercises in the context of the Gaza strip is crucial. Due to the potential for escalation of conflict in selected areas, this extra allocation in relation to logistical support and timeframe mitigated any major impact on the actual timely conclusion of the assessment. Data collection was halted for 2 days due to the escalation of the conflict that affected the selected areas, and the buffer days were used to complete the data collection. Allocation of extra days meant ensuring that budget, cars, and other logistical support was available in a timely manner and did not require extra approvals and procedures.
- *In country and remote assistance* to the data collection teams were provided by all the agencies involved during the data collection but mostly during data processing and analysis. The lead researcher could not visit all the areas and during the potential escalation of the conflict could not visit the field and support the data collection teams

## Conclusions

Despite the documented challenges, this research has highlighted the need to assess the way breastfeeding is protected and supported in the Gaza strip, and possibly to strengthen the capacity of midwives in providing breastfeeding support, including the timely initiation of breastfeeding and lactation support. Indeed, the protracted emergency settings may have limited midwives’ ability to access professional updates and on the job capacity building opportunities. Implementing partners must address this gap in knowledge and skill as a matter of urgency.

The role of midwives in supporting women to improve their breastfeeding practices has several important effects on maternal health and wellbeing and proven positive effects for children. In humanitarian contexts such as the Gaza strip, the initiation and continuation of effective breastfeeding has lifesaving impacts. This study raises alerts humanitarian agencies on the importance of the need to invest more in evidence-based interventions and the capacity of midwives in humanitarian contexts. If this need is met, midwives’ effective support of improved breastfeeding practices will contribute to reducing pressure on Gaza’s public health system, currently stretched in a fragile and constant crisis setting.

The study in Gaza highlights the importance of supporting research in protracted crisis settings. It proves that it is possible to conduct research in complex humanitarian environments. The value of this study is linked to its context and the living conditions of the most vulnerable communities in the Gaza strip. Lessons learned in this research include the importance of a) allocating additional research time, to account for potential interruption, due to the volatile situation b) daily coordination with security officers to ensure safe access to target localities c) identification of extra sites, in case escalation of conflicts do not allow for access to pre-selected localities and d) training of additional enumerators to fill up for potential gaps due to the conflict escalation and e) negotiation with the authorities to ensure compliance with the requirements. Finally, it was observed that there is a general need for health and nutrition workers to increase their knowledge and skills on nutrition assessments.

## Data Availability

The datasets generated and/or analysed during the current study are not publicly available due to the specific operational context but are available from the corresponding author on reasonable request and upon approval from the leading agencies.

## Abbreviations

ARI: Acute Respiratory Infection
CS: Caesarean
ENA: Emergency Nutrition Assessment
IYCF: Infant and Young Child Feeding
IYCF-E: Infant and Young Child Feeding in emergencies
KAP: Knowledge, Attitudes and Practices
MEAL: Monitoring, Evaluation, Accountability and Learning
MICS: Multi Indicator Cluster Survey
OCHA: United Nations Office for the Coordination of Humanitarian Affairs
oPt: Occupied Palestinian Territory
SCI: Save the Children International Occupied Palestinian Territory
SMART: Standardized Monitoring and Assessment of Relief and Transitions
UNICEF: United Nations Children’s Funds
UN: United Nations
UK: United Kingdom
WASH: Water, Sanitation and Hygiene
WFP: World Food Programme

## References

[1] Victora CG, Bahl R, Barros AJ, França GV, Horton S, Krasevec J (2016). Breastfeeding in the 21st century: epidemiology, mechanisms, and lifelong effect. Lancet.

[2] Jones G, Steketee RW, Black RE, Bhutta ZA, Morris SS (2003). Bellagio child survival study group. How many child deaths can we prevent this year?. Lancet..

[3] WHO (2013). Long term effects of breastfeeding.

[4] Ip S, Chung M, Raman G, Trikalinos TA, Lau J. A summary of the Agency for Healthcare Research and Quality's evidence report on breastfeeding in developed countries. Breastfeed Med. 2009;4 Suppl 1:S17-30. 10.1089/bfm.2009.0050. 10.1089/bfm.2009.0050 19827919

[5] Swerts M, Westhof E, Bogaerts A, Lemiengre J (2016). Supporting breast-feeding women from the perspective of the midwife: a systematic review of the literature. Midwifery..

[6] McFadden A, Gavine A, Renfrew MJ, Wade A, Buchanan P, Taylor JL, Veitch E, Rennie AM, Crowther SA, Neiman S, MacGillivray S. Support for healthy breastfeeding mothers with healthy term babies. Cochrane Database Syst Rev. 2017;2(2):CD001141. 10.1002/14651858.CD001141.pub5. 10.1002/14651858.CD001141.pub5 PMC6464485 28244064

[7] UN OCHA (2019). Palestine, humanitarian needs overview.

[8] UNRWA. Web report. Available from https://www.unrwa.org/activity/health-gaza-strip. [Accessed 10 Feb 2020].

[9] WHO (2019). Health conditions in the occupied Palestinian territory, including east Jerusalem, and in the occupied Syrian Golan.

[10] UNICEF. Health and nutrition profile, Palestine. Available from https://www.unicef.org/sop/what-we-do/health-and-nutrition. [Accessed 10 Mar 2020].

[11] UNICEF and PCBS (2014). Multiple indicator cluster survey state of Palestine.

[12] Palestinian Central Bureau of Statistics (2017). Census.

[13] Save the Children, WFP, UNICEF (2018). The multi-sectoral nutrition assessment conducted in the vulnerable areas of the Gaza Strip, occupied Palestinian territory, from October 15 to 31, 2018.

[14] Jakobsen M, Sodemann M, Nylén G, Balé C, Nielsen J, Lisse I, Aaby P (2003). Breastfeeding status as a predictor of mortality among refugee children in an emergency situation in Guinea-Bissau. Tropical Med Int Health.

[15] Black RE, Allen LH, Bhutta ZA, Caulfield LE, de Onis M, Ezzati M, for the maternal and child Undernutrition study group* (2008). Maternal and child undernutrition: global and regional exposures and health consequences. Lancet.

[16] Anderson N (2010). Breast-feeding in a complex emergency: four linked cross-sectional studies during the Bosnian conflict. Public Health Nutr.

[17] Creek T, Kim A, Lu L (2007). Role of infant feeding and HIV in a severe outbreak of diarrhoea and malnutrition among young children, Botswana, 2006 Paper presented at: 14th conference on retroviruses and opportunistic infections.

[18] Hipgrave DB, Assefa F, Winoto A, Sukotjo S (2012). Donated breast milk substitutes and incidence of diarrhoea among infants and young children after the May 2006 earthquake in Yogyakarta and Central Java. Public Health Nutr.

[19] Ishii K, Goto A, Ota M, Yasumura S, Abe M, Fujimori K (2016). Factors associated with infant feeding methods after the nuclear power plant accident in Fukushima: data from the pregnancy and birth survey for the fiscal year 2011 Fukushima health management survey. Matern Child Health J.

[20] Slade AC (2015). Shaken but not broken: supporting breastfeeding women after the 2011 Christchurch New Zealand earthquake. Breastfeed Rev.

[21] DeYoung SE, Chase J, Branco MP, Park B (2018). The effect of mass evacuation on infant feeding: the case of the 2016 Fort McMurray Wildfire. Matern Child Health J.

[22] Bäckström CA, Wahn EIH, Ekström AC (2010). Two sides of breastfeeding support: experiences of women and midwives. Int Breastfeed J.

[23] Raisler J (2000). Midwives helping mothers to breastfeed: food for thought and action. J Midwifery Women Health.

[24] ENN, IFE Core Group (2017). Infant and young child feeding in emergencies. Operational guidance for emergency relief staff and programme managers.

[25] WHO (2009). Infant and young child feeding. Model chapter for textbooks for medical students and allied professions.

[26] WHO, UNICEF (2018). Baby friendly hospital Initiative recommendations.

