# Correction to: Breastfeeding knowledge of mothers in protracted crises: the Gaza Strip example

**DOI:** 10.1186/s12889-021-10918-2

**Published:** 2021-05-12

**Authors:** Alessandro Iellamo, Emily Monaghan, Samar A. L. Moghany, Jonathan Latham, Nihal Nassereddin

**Affiliations:** 1 Save the Children, London, UK; 2 Save the Children International, Occupied Palestine Territory, Gaza, Palestine; 3 World Food Programme Palestine, Jesuralem, Israel

**Correction to: BMC Public Health 21, 742 (2021)**

**https://doi.org/10.1186/s12889-021-10748-2**

It was highlighted that in the original article [1] the given name and family name of the first author, Alessandro Iellamo, were interchanged. The original article has been updated.
